# Retention of the posterior cruciate ligament versus the posterior stabilized design in total knee arthroplasty: a prospective randomized controlled clinical trial

**DOI:** 10.1186/1471-2474-10-119

**Published:** 2009-09-30

**Authors:** Lennard GH van den Boom, Reinoud W Brouwer, Inge van den Akker-Scheek, Sjoerd K Bulstra, Jos JAM van Raaij

**Affiliations:** 1 Department of Orthopaedic Surgery, Martini Hospital, van Swietenplein 1, 9728NT, Groningen, The Netherlands; 2 Department of Orthopaedic Surgery, University Medical Center Goningen, University of Groningen, PO Box 30.001, 9700 RB, Groningen, The Netherlands

## Abstract

**Background:**

Prosthetic design for the use in primary total knee arthroplasty has evolved into designs that preserve the posterior cruciate ligament (PCL) and those in which the ligament is routinely sacrificed (posterior stabilized). In patients with a functional PCL the decision which design is chosen depends largely on the favour and training of the surgeon.

The objective of this study is to determine whether the patient's perceived outcome and speed of recovery differs between a posterior cruciate retaining total knee arthroplasty and a posterior stabilized total knee arthroplasty.

**Methods/Design:**

A randomized controlled trial will be conducted. Patients who are admitted for primary unilateral TKA due to primary osteoarthrosis are included when the following inclusion criteria are met: non-fixed fixed varus or valgus deformity less than 10 degrees, age between 55 and 85 years, body mass index less than 35 kg/m^2 ^and ASA score (American Society of Anaesthesiologists) I or II. Patients are randomized in 2 groups. Patients in the posterior cruciate retaining group will receive a prosthesis with a posterior cut-out for the posterior cruciate ligament and relatively flat topography. In patients allocated to the posterior stabilized group, in which the posterior cruciate ligament is excised, the design may substitute for this function by an intercondylar tibial prominence that articulates with the femur in flexion. Measurements will take place preoperatively and 6 weeks, 3 months, 6 months and 1 year postoperatively.

At all measurement points patient's perceived outcome will be assessed using the Western Ontario and McMaster Universities Osteoarthritis Index (WOMAC). Secondary outcome measures are quality of life (SF-36) and physician reported functional status and range of motion as determined with the Knee Society Clinical Rating System (KSS).

**Discussion:**

In the current practice both posterior cruciate retaining and posterior stabilized designs for total knee arthroplasty are being used. To date no studies have been performed determining whether there is a difference in patient's perceived outcome between the two designs. Additionally, there is a lack of studies determining the speed of recovery in both designs as most studies only determine the final outcome. This randomised controlled study has been designed to determine whether the patient's perceived outcome and speed of recovery differs between a posterior cruciate retaining total knee arthroplasty and a posterior stabilized total knee arthroplasty.

**Trial Registration:**

The trial is registered in the Netherlands Trial Registry (NTR1673).

## Background

Prosthetic design for the use in primary total knee arthroplasty has evolved into designs that preserve the posterior cruciate ligament (PCL) and those in which the ligament is routinely sacrificed (posterior stabilized). However, merely saving the PCL does not provide for normal knee kinematics unless a proper design is used and surgical technique is precise [1]. Posterior stabilized implants in which the ligament is excised may substitute for this function by an intercondylar tibial prominence that articulates with the femur in flexion, aiding in femoral roll-back [2]. In femoral roll-back there is a relative internal tibial rotation with flexion as the lateral condyle moves more posteriorly due to less constraint [3]. This femoral roll-back accounts for moving the tibiofemoral contact posteriorly by approximately 10 mm and represents approximately a 30 percent change in the lever arm of the quadriceps mechanism. This possible advantage is not present in the PCL-substituting designs [4].

In the current practice both designs are used. In patients whith a non functional PCL the posterior stabilised design is used. However, in patients with a functional PCL the decision which design is chosen depends largely on the favour and training of the surgeon. A limited amount of studies have been performed into the difference in outcome of the two designs. These studies are characterised by a small amount of patients, different outcome measures, poor randomisation and comparing designs of different manufacturers [5]. Range of motion was the only common outcome parameter; a meta-analysis showed a difference in range of motion and reproduction angle favouring posterior stabilized designs over PCL retention designs one year postoperatively. However, it is uncertain whether this observation is of clinical relevance [5]. To date, no studies have been performed determining whether there is a difference between designs in patient's perceived outcome. Additionally, there is a lack of studies determining the speed of recovery in both designs as most studies only determine the final outcome (e.g. after one year).

The primary objective of this randomized controlled trial is to examine whether there is a difference in patient's perceived outcome as determined with The Western Ontario and McMaster Universities Osteoarthritis Index (WOMAC) between a patient group receiving a posterior cruciate retaining total knee arthroplasty compared to a group receiving a posterior stabilized total knee arthroplasty.

## Methods

### Study design

A randomized controlled trial will be conducted. Patients that are scheduled for a primary total knee arthroplasty and apply to the inclusion criteria of the study are informed about the trial. After consent to participate, patients are allocated to receive either a posterior cruciate retaining total knee arthroplasty or a posterior stabilized total knee arthroplasty. The randomization procedure is based on sequentially numbered opaque sealed envelopes, produced by an external institution not involved in the selection, clinical care and evaluation of the patients. Two orthopaedic surgeons will perform the surgical procedure, therefore, block randomisation is used. The study design, procedures and informed consent are approved by the local Medical Ethical Committee (registration number 2007-23). The trial is registered in the Netherlands Trial Registry (NTR1673).

### Study population

The study will be conducted at the Department of Orthopaedic Surgery of the Martini Hospital, which is a large teaching hospital in the city of Groningen, the Netherlands. Patients who are admitted for primary unilateral TKA due to primary osteoarthrosis are included when the following inclusion criteria are met: non-fixed fixed varus or valgus deformity less than 10 degrees, age between 55 and 85 years, body mass index less than 35 kg/m^2 ^and ASA score (American Society of Anaesthesiologists) I or II.

Patients with secondary osteoarthritis of the knee, rheumatic disease, a flexion less than 90 degrees, a flexion contracture over 10 degrees, peripheral neuropathy or a history of a cerebral vascular accident are excluded. Participation in the study is voluntary and informed consent is required. The inclusion period is planned from January 2008 to January 2010.

If perioperatively an insufficient posterior cruciate ligament is found, the patient will receive a posterior stabilized total knee prosthesis. Analyses will be done according to the intention-to-treat principle, with a sub-analysis excluding these conversions. As the incidence of an insufficient posterior cruciate ligament is higher in patients with secondary osteoarthritis of the knee, rheumatic diseases and a fixed varus or valgus deformity of over 10 degrees, these are exclusion criteria of our study.

### Intervention

The Anatomic Graduated Component (AGC; Biomet, Inc, Warsaw, IN, USA) is implanted, either the posterior cruciate retaining or the high post posterior stabilized design.

The AGC has been succesfully used in our hospital for over 20 years. We specifically choose this implant since we have very good experiences with it, with a high survival rate. Wordlwide it has been implanted since 1983 with 95%-98% survivorship at 15 years. Several AGC studies have demonstrated these results [6-8].

The AGC consists of a one-piece molded polyethylene tibial component with a durable cobalt chrome femoral component. Regarding the differences between the AGC designs, the posterior cruciate retaining (PCR) design has a posterior cut-out for the posterior cruciate ligament and relatively flat topography. This allows for posterior roll-back of the femur when the knee is flexed and the posterior cruciate ligament is tensioned.

In the posterior stabilized (PS) design the stabilization is achieved by a "cam and post" mechanism added to the prosthesis components. This mechanism replaces the function of the posterior criciate ligament. The femoral component has a transverse cam added to the backside of the prosthesis. The tibial polyethylene plate has a central polyethylene post placed on the middle of the plate. In the assembled total knee, the cylindrical cam comes against the post when the total knee bends. The postthen forces the cam backwards preventing the forward glide of the femoral component. In this way the posterior stabilized total knee replaces the function of the posterior cruciate ligament.

PCR and PS designs from other implants are based on the same biomechanical principles as described above, although there might be some slight differences in design, surgical technique and materials used among different manufacturers. One main advantage of our study, and in which our study differs from others, is that we compare one AGC design with another design of the same implant, which minimizes the variables that differ between the two groups of patients, especially with respect to surgical technique and materials used.

The surgical procedure consists of a midline approach. After the midline skin incision has been made over the knee joint, the capsule of the joint is exposed. By incising the joint capsule, the anteromedial arthrotomy is performed. The capsule is incised anteriorly, just superior to the patella, curving medially from the patella and back down anteriorly, just medial from the patellar ligament.

Antibiotic prophylaxis with a first-generation cephalosporin will be given preoperatively and during the first twenty-four hours intravenously All patients will be treated with the same, standardized protocol postoperatively, in terms of analgesia and mobilization. Subcutaneous low molecular weight heparin is given for 6 weeks postoperatively as prophylaxis against thrombosis. At day one after surgery, physiotherapy is started and patients are mobilized with the aid of two crutches. When patients are able to walk stairs and make transfers to bed and toilet, and when the knee flexes at least 90 degrees, they are discharged from the hospital. Physiotherapy will be continued in the home situation for another 6 weeks.

### Measurements

Measurements will take place preoperatively and 6 weeks, 3 months, 6 months and 1 year postoperatively. In this study the primary outcome parameter is the patient's perceived outcome which will be determined with the Western Ontario and McMaster Universities Osteoarthritis Index (WOMAC). The WOMAC is the most frequently used and recommended questionnaire to determine outcome after TKA [9,10]. The Dutch version has proven to be reliable and valid [11].

Secondary outcome parameters. Physician reported functional status, including range of motion, will be measured with the validated Knee Society Clinical Rating System (KSS) [12]. The 36-item Short Form Health Survey (SF-36) Dutch language version will be used to assess the health related quality of life [13]. Moreover, surgical approach, surgical time and intra-operative blood loss will be recorded and perioperative complications will be registered. The study will be double blinded: the KSS will be completed by a blinded independent observer and the patient will not be informed about which design is used during the length of the study.

### Sample Size

Sample size calculation is based on the primary outcome measure (WOMAC). A difference between the two groups of 15% is considered clinically relevant. With a standard deviation of 19.0 points, α = 0.05 and power of 80%, 55 patients are needed in each group. With an estimated drop-out of 10% in total 120 patients are needed, 60 patients in each group.

### Statistical analysis

Descriptive statistics (means and standard deviations) will be used to describe the patient characteristics and outcome variables at the 5 measurement points.

A GLM repeated measures analysis will be used to determine whether there is a difference on the primary and secondary outcome variables between the two groups over time. A p-value of < 0.05 is considered significant.

## Discussion

In the current practice both posterior cruciate retaining and posterior stabilized designs for total knee arthroplasty are being used. In patients with a functional PCL the decision which design is chosen depends largely on the favour and training of the surgeon. To date, no studies have been performed determining whether there is a difference between designs in patient's perceived outcome. Additionally, there is a lack of studies determining the speed of recovery in both designs as most studies only determine the final outcome (e.g. after one year). This study will overcome these drawbacks.

## Competing interests

The authors will not receive any reimbursements, fees or salary for performing the study.

## Authors' contributions

LGHVDB designed the study, coordinates the trial, wrote the manuscript, and will analyze and report the data. IVDAS designed the study, coordinates the trial and will review all patients. JJAMVR, RWB and SKB designed the study, and JJAMVR and RWB will include and operate all patients. All authors read, edited and approved the final manuscript.

## Pre-publication history

The pre-publication history for this paper can be accessed here:


